# A National Survey of Marijuana Use Among US Adults With Medical Conditions, 2016-2017

**DOI:** 10.1001/jamanetworkopen.2019.11936

**Published:** 2019-09-20

**Authors:** Hongying Dai, Kimber P. Richter

**Affiliations:** 1 College of Public Health, University of Nebraska Medical Center, Omaha; 2 Department of Population Health, University of Kansas Medical Center, Kansas City

## Abstract

**Importance:**

The number of states legalizing marijuana for medical and recreational use is increasing. Little is known regarding how or why adults with medical conditions use it.

**Objectives:**

To report the prevalence and patterns of marijuana use among adults with and without medical conditions, overall and by sociodemographic group, and to further examine the associations between current marijuana use and the types and number of medical conditions.

**Design, Setting, and Participants:**

This survey study used a probability sample of US adults aged 18 years and older from the 2016 and 2017 Behavioral Risk Factor Surveillance System, a telephone-administered survey that collects data from a representative sample of US adult residents across the states regarding health-related risk behaviors, chronic health conditions, and use of preventive services.

**Main Outcomes and Measures:**

Current (past month) and daily (≥20 days in the last 30 days) marijuana use.

**Results:**

The study sample included 169 036 participants (95 780 female [weighted percentage, 52.0%]). Adults with medical conditions had higher odds of reporting current marijuana use than those without medical conditions (age 18-34 years: adjusted odds ratio, 1.8 [95% CI, 1.5-2.1]; age 35-54 years: adjusted odds ratio, 1.4 [95% CI, 1.2-1.7]; age ≥55 years: adjusted odds ratio, 1.6 [95% CI, 1.3-2.0]), especially among those with asthma, chronic obstructive pulmonary disease, arthritis, cancer, and depression. Among those with medical conditions, the prevalence of marijuana use decreased with increasing age, ranging from 25.2% (95% CI, 22.0%-28.3%) for those aged 18 to 24 years to 2.4% (95% CI, 2.0%-2.8%) for those aged 65 years or older for current marijuana use and from 11.2% (95% CI, 8.7%-13.6%) to 0.9% (95% CI, 0.7%-1.2%), respectively, for daily marijuana use. Most adults who used marijuana (77.5%; 95% CI, 74.7%-80.3%), either with or without medical conditions, reported smoking as their primary method of administration. Adults with medical conditions were more likely than those without medical conditions to report using marijuana for medical reasons (45.5% [95% CI, 41.1%-49.8%] vs 21.8% [95% CI, 17.8%-25.7%]; difference, 23.7% [95% CI, 17.8%-29.6%]) and less likely to report using marijuana for recreational purposes (36.2% [95% CI, 32.1%-40.3%] vs 57.7% [95% CI, 52.6%-62.9%]; difference, −21.5% [95% CI, −28.1% to 14.9%]).

**Conclusions and Relevance:**

This study found that marijuana use was more common among adults with medical conditions than those without such conditions. Notably, 11.2% of young adults with medical conditions reported using marijuana on a daily basis. Clinicians should screen for marijuana use among patients, understand why and how patients are using marijuana, and work with patients to optimize outcomes and reduce marijuana-associated risks.

## Key Points

**Question:**

What are the prevalence and patterns of marijuana use among adults with medical conditions?

**Findings:**

This survey study using data from 169 036 participants in the 2016 and 2017 Behavioral Risk Factor Surveillance System surveys found that, compared with adults without medical conditions, adults with medical conditions had a significantly higher prevalence of current and daily marijuana use, were more likely to report using marijuana for medical reasons, and were less likely to report using marijuana for recreational purposes. Among respondents with medical conditions, 11.2% of young adults reported using marijuana on a daily basis, and the prevalence of marijuana use decreased with increasing age.

**Meaning:**

Clinicians should discuss marijuana use with their patients to optimize medical outcomes.

## Teaser

This survey study analyzes the prevalence and patterns of marijuana use among adults with and without medical conditions using data from the 2016 and 2017 Behavioral Risk Factor Surveillance System surveys.

## Introduction

Public opinion on marijuana has changed dramatically over the last 2 decades. Support for legalization has doubled since 2010, and currently, 62% of US adults support marijuana use.^1^ Although marijuana is still classified as a schedule I drug at the federal level, as of June 2019, 33 states and the District of Columbia have legalized 1 or more forms of marijuana; 11 states and the District of Columbia have approved marijuana for both medical and recreational uses.^2^ In the meantime, current (past-month) marijuana use has increased from 6.2% in 2002 to 9.6% in 2017 among persons aged 12 years or older in the United States.^3^ In 2017, 24.4 million US adults aged 18 years or older were current users of marijuana; young adults aged 18 to 25 years had the highest prevalence (22.7%).^3^

Although much policy change has focused on the medical use of marijuana, very little is known regarding whether or how it is actually used for medical purposes, including whether patients are using nonprescribed marijuana for medical purposes (ie, self-medicating) or obtaining marijuana in accordance with physician recommendations. Few studies have examined the characteristics of marijuana users and the prevalence of use among populations with different medical conditions. Those who use marijuana believe that its benefits include pain management, ameliorating chronic conditions such as epilepsy and multiple sclerosis, and relieving anxiety, stress, and depression.^4^ Current research suggests that both short- and long-term marijuana use are associated with several adverse health outcomes, including respiratory symptoms, cognitive decline, neurological changes, and psychiatric conditions including addiction.^5^ Other potential long-term health consequences include cancer, chronic obstructive pulmonary disease (COPD), and heart disease.^5^

A previous study^6^ found that individuals who used marijuana in the past year were less likely than nonmarijuana users to have diabetes but more likely to have depression. The prevalence of marijuana use was not significantly different among those with and without multiple medical conditions.^6^ However, that study was limited to marijuana use of middle-aged and older adults (ie, those aged ≥50 years) in the past year.

The fundamental pattern of the use of marijuana among patients with medical conditions remains unknown. Hence, it is not clear how many patients with medical conditions are using marijuana or how they are using it, and this is a critical knowledge gap. Clinicians should know whether marijuana use is prevalent among their patients with chronic illness, to implement screening and counseling regarding potential health risks and benefits. Policy makers should know whether highly vulnerable patient populations are using marijuana to determine whether heightened surveillance is needed to determine the associations of marijuana use with medical care and outcomes.

To begin to address this knowledge gap, we used a probability sample by combining the 2016 and 2017 Behavioral Risk Factor Surveillance System (BRFSS) surveys—the nation’s largest health survey—to assess the prevalence of current (past-month) and daily (≥20 days in the last 30 days) marijuana use across key sociodemographic groups. We further examined the associations between current marijuana use and the types and number of medical conditions, stratified by 3 age groups (18-34, 35-54, and ≥55 years). This study also determined the method of administration by which participants primarily use marijuana and assessed whether they used marijuana for medical or recreational purposes.

## Methods

### Data

The BRFSS is a telephone-administered survey that collects data from a representative sample of US adult residents across the states regarding health-related risk behaviors, chronic health conditions, and use of preventive services. The survey uses a random digital dialing technique with no incentive for participation.^7^ The BRFSS completes more than 400 000 adult interviews each year, and the combined 2016 and 2017 BRFSS included a total of 936 945 respondents (486 303 in 2016 and 450 642 in 2017). The overall median response rates for participating states and territories were 47.1% and 45.9% for the 2016 and 2017 BRFSS, respectively.^8^ Survey items on marijuana use were first added to the BRFSS in 2016 as an optional module in select US states and territories (Table 1). Our analysis includes respondents who were administered the marijuana use module in the 2016 and 2017 BRFSS surveys. Informed consent was obtained from all participants before the interview. A detailed description of the BRFSS survey design, questionnaires, and data collection can be found on the BRFSS website.^7,9^ Because the BRFSS provides publicly available deidentified data, this study was determined to be nonhuman subjects research by the University of Nebraska Medical Center institutional review board.

**Table 1.** Prevalence of Current and Daily Marijuana Use in Select Geographic US Regions by Medical Condition Using Combined 2016 and 2017 Behavioral Risk Factor Surveillance System Surveys^a^

Characteristic	Current Marijuana Use				Daily Marijuana Use			
	Weighted % (95% CI)^b^		*P* Value^c^	Adjusted *P* Value^d^	Weighted % (95% CI)^b^		*P* Value^c^	Adjusted *P* Value^d^
	No Medical Condition (n = 64 808)	Medical Condition (n = 104 228)			No Medical Condition (n = 64 808)	Medical Condition (n = 104 228)		
Overall	8.3 (7.8-8.8)	8.8 (8.3-9.2)	.21	<.001	3.6 (3.3-4.0)	3.9 (3.6-4.3)	.27	<.001
Sex								
Male	11.3 (10.5-12.1)	11.1 (10.4-11.9)	.76	<.001	5.4 (4.8-6.0)	5.2 (4.7-5.7)	.62	<.001
Female	5.0 (4.4-5.6)	6.9 (6.3-7.5)	<.001	<.001	1.7 (1.4-2.1)	2.9 (2.5-3.4)	<.001	<.001
Race/ethnicity								
Non-Hispanic white	8.9 (8.2-9.6)	8.3 (7.8-8.8)	.14	<.001	4.0 (3.5-4.5)	3.7 (3.3-4.0)	.27	<.001
Non-Hispanic black	10.7 (9.1-12.4)	9.9 (8.3-11.6)	.49	.04	5.5 (4.2-6.7)	4.8 (3.5-6.1)	.50	.39
Hispanic	6.3 (5.2-7.3)	9.3 (7.7-10.9)	.001	<.001	2.4 (1.7-3.1)	4.1 (2.9-5.3)	.01	<.001
Other	7.3 (5.5-9.1)	11.5 (8.8-14.2)	.008	<.001	2.5 (1.6-3.5)	4.9 (2.7-7.1)	.02	<.001
Education								
Less than high school	7.5 (5.5-9.5)	7.4 (6.3-8.5)	.95	.02	3.6 (2.4-4.8)	3.6 (2.8-4.4)	.97	.04
High school graduate	9.1 (8.2-10.1)	8.9 (8.0-9.8)	.77	<.001	4.5 (3.8-5.2)	4.5 (3.7-5.2)	.91	<.001
Some college	10.6 (9.5-11.6)	10.8 (9.8-11.8)	.81	<.001	4.7 (3.9-5.4)	4.8 (4.1-5.5)	.80	<.001
College graduate	5.6 (5.0-6.2)	6.7 (6.0-7.4)	.02	<.001	1.8 (1.5-2.1)	2.3 (1.9-2.6)	.07	.001
Income, $US								
<25 000	8.8 (7.7-9.9)	10.1 (9.3-11.0)	.06	<.001	4.3 (3.5-5.2)	4.7 (4.1-5.3)	.53	.002
25 000-49 999	9.3 (8.3-10.4)	7.6 (6.8-8.4)	.01	.005	4.3 (3.6-5.1)	3.8 (3.2-4.4)	.28	<.001
50 000-74 999	8.3 (6.9-9.7)	8.8 (7.5-10.0)	.63	<.001	4.0 (3.0-4.9)	4.1 (3.1-5.0)	.83	.001
≥75 000	8.3 (7.2-9.3)	9.3 (8.2-10.5)	.17	<.001	3.1 (2.5-3.8)	3.7 (2.9-4.5)	.29	.002
Employment status								
Employed	9.1 (8.4-9.7)	10.9 (10.1-11.7)	<.001	<.001	4.1 (3.7-4.6)	5.1 (4.6-5.6)	.006	<.001
Unemployed	11.4 (9.1-13.8)	16.3 (13.8-18.8)	.006	<.001	5.6 (3.9-7.4)	7.0 (5.4-8.6)	.26	.08
Not in workforce	5.6 (4.8-6.4)	6.2 (5.6-6.8)	.30	<.001	2.0 (1.4-2.5)	2.6 (2.1-3.1)	.07	<.001
Home ownership								
Own	6.1 (5.5-6.7)	6.3 (5.7-6.8)	.66	<.001	2.5 (2.2-2.9)	2.4 (2.1-2.8)	.73	.008
Rent	12.1 (11.1-13.2)	15.1 (14.1-16.2)	<.001	<.001	5.6 (4.8-6.4)	7.9 (7.1-8.7)	<.001	<.001
Other	13.3 (10.8-15.8)	15.1 (12.6-17.7)	.32	.006	5.8 (3.8-7.8)	6.6 (4.8-8.5)	.57	.13
Marijuana legalization status^e^								
No	6.9 (6.4-7.4)	6.3 (5.9-6.7)	.05	<.001	3.2 (2.8-3.6)	2.8 (2.5-3.1)	.06	<.001
Medical	6.1 (5.5-6.6)	7.1 (6.5-7.7)	.01	<.001	2.5 (2.2-2.9)	2.7 (2.3-3.0)	.66	.009
Recreational	11.3 (10.0-12.6)	14.2 (12.9-15.5)	.003	<.001	4.7 (3.9-5.6)	6.6 (5.6-7.5)	.005	<.001

^a^ In 2016, 10 states (Alaska, Colorado, Florida, Idaho, Minnesota, Mississippi, Nebraska, Ohio, Tennessee, and Wyoming) participated in the optional marijuana use module. In 2017, 9 states (Alaska, California, Georgia, Idaho, Minnesota, New Hampshire, South Carolina, Tennessee, and Wyoming) and 2 territories (Guam and Puerto Rico) participated in the optional marijuana use module.

^b^ Weighted percentages and 95% CIs were reported by taking the complex sampling design into account.

^c^ A Rao-Scott χ^2^ test was performed for the univariate analysis of the difference in marijuana use by medical condition.

^d^ Multivariable logistic regression was performed for the multivariate analysis of the difference in marijuana use by medical condition. The analysis was adjusted by age, sex, race/ethnicity, education, income, survey year, and state.

^e^ In 2016, medical marijuana use was legal in Minnesota and recreational use was legal in Alaska and Colorado. In 2017, medical use was legal in Minnesota, and recreational use was legal in Alaska and California.

### Measures

#### Marijuana Use

Marijuana use was assessed by the question, “During the past 30 days, on how many days did you use marijuana or hashish?” Those who responded that they used it 1 day or more were categorized as current marijuana users, and those who responded that they used it 20 to 30 days were categorized as daily marijuana users.^10^ Of the 170 271 respondents who were administered marijuana use questions, we excluded those who answered “don’t know or not sure” (515 participants) or refused to answer (720 participants).

#### Method of Administration and Reasons to Use Marijuana

Method of administration was assessed by the question, “During the past 30 days, how did you primarily use marijuana?” with response options of “smoke it (for example, in a joint, bong, pipe, or blunt),” “eat it (for example, in brownies, cakes, cookies, or candy),” “drink it (for example, in tea, cola, or alcohol),” “vaporize it (for example, in an e-cigarette-like vaporizer),” “dab it (for example, using butane hash oil, wax, or concentrates),” and “use it some other way.” Because the 2016 BRFSS item directed participants to endorse all that apply, whereas the 2017 BRFSS item directed participants to provide only 1 answer, we limited our analyses of the method of administering marijuana to the 2017 BRFSS data. We classified the participants into 6 groups: smoke it, eat it, vaporize it, drink it, dab it, and other way.

An item assessing reasons for using marijuana was included only in the 2017 BRFSS. It was assessed by the question, “When you used marijuana or hashish during the past 30 days, was it for medical reasons to treat or decrease symptoms of a health condition, or was it for non-medical reasons to get pleasure or satisfaction (such as: excitement, to ‘fit in’ with a group, increased awareness, to forget worries, for fun at a social gathering)?” Participants were classified into 3 groups on the basis of their response: medical reasons, nonmedical reasons, and both.

#### Medical Comorbidity

Chronic health conditions were assessed by the question, “Has a doctor, nurse, or other health professional ever told you that you had any of the following?” with answers including stroke, heart attack, angina or coronary heart disease, asthma, COPD, diabetes, arthritis, kidney disease, skin cancer, depressive disorder, and other types of cancer. Those who responded as having at least 1 chronic health condition were classified as having medical conditions.

#### Covariates

Several covariates were included in the analysis to adjust for confounding influences. These covariates included sex (male and female), race/ethnicity (non-Hispanic white, non-Hispanic black, Hispanic, and non-Hispanic other), education level (less than high school, high school graduate, some college, or college graduate), annual income (<$25 000, $25 000-$49 999, $50 000-74 999, ≥$75 000), and age (18-24, 25-34, 35-44, 45-54, 55-64, and ≥65 years).

### Statistical Analysis

Weighted estimates of current and daily marijuana use by medical condition were calculated by taking the survey stratum and sampling weights into account, both overall and by sociodemographic factors, for the combined 2016 and 2017 BRFSS data. Group differences between those with and without medical conditions were detected by a Rao-Scott χ^2^ test in the univariate analysis and the logistic regression model in the multivariable analysis. We further performed separate logistic regressions to examine the associations between marijuana use and the types and number of medical conditions. In the multivariable analysis, sex, age, education, income, race/ethnicity, and state were included as covariates. The 2017 BRFSS data were used to report the method of administration and reasons to use marijuana among current marijuana users. Statistical analyses were performed using SAS statistical software version 9.4 (SAS Institute), and 2-tailed *P* < .05 was considered statistically significant.

## Results

The study sample included 169 036 participants from the combined 2016 and 2017 BRFSS surveys (95 780 female [weighted percentage, 52.0%]; non-Hispanic white, 60.9%; non-Hispanic black, 11.0%; Hispanic, 19.9%; college graduate, 26%; annual income ≥$50 000, 46.7%). Overall, 53.7% of adults reported at least 1 medical comorbidity. Compared with those without medical conditions, adults who reported any medical conditions were more likely to be older, female, non-Hispanic white, have less than a high school education, have annual income less than $15 000, and own a home and were less likely to be employed. The eTable in the Supplement compares sociodemographic characteristics of adults with and without medical conditions.

Table 1 presents the prevalence of current and daily marijuana use by medical condition and sociodemographic characteristics. Overall, 8.8% (95% CI, 8.3%-9.2%) of adults with medical conditions reported current marijuana use, and 3.9% (95% CI, 3.6%-4.3%) reported daily marijuana use. Among adults without medical conditions, 8.3% (95% CI, 7.8%-8.8%) reported current marijuana use and 3.6% (95% CI, 3.3%-4.0%) reported daily use. In the univariate analysis, current marijuana use among adults with medical conditions (vs those without medical conditions) was higher among women (6.9% [95% CI, 6.3%-7.5%] vs 5.0% [95% CI, 4.4%-5.6%]; difference, 1.9% [95% CI, 1.1%-2.8%]), Hispanic individuals (9.3% [95% CI, 7.7%-10.9%] vs 6.3% [95% CI, 5.2%-7.3%]; difference, 3.1% [95% CI, 1.2%-5.0%]) and those of other races/ethnicities (nonwhite, nonblack, and non-Hispanic, 11.5% [95% CI, 8.8%-14.2%] vs 7.3% [95% CI, 5.5%-9.1%]; difference, 4.2% [95% CI, 1.0%-7.5%]), college graduates (6.7% [95% CI, 6.0%-7.4%] vs 5.6% [95% CI, 5.0%-6.2%]; difference, 1.1% [95% CI, 0.2%-2.0%]), those employed (10.9% [95% CI, 10.1%-11.7%] vs 9.1% [95% CI, 8.4%-9.7%]; difference, 1.9% [95% CI, 0.8%-2.9%]), those who were unemployed (16.3% [95% CI, 13.8%-18.8%] vs 11.4% [95% CI, 9.1%-13.8%]; difference, 4.9% [95% CI, 1.5%-8.2%]), renters (15.1% [95% CI, 14.1%-16.2%] vs 12.1% [95% CI, 11.1%-13.2%]; difference, 3.0% [95% CI, 1.5%-4.5%]), and those residing in states where marijuana was legal for either medical (7.1% [95% CI, 6.5%-7.7%] vs 6.1% [95% CI, 5.5%-6.6%]; difference, 1.0% [95% CI, 0.2%-1.8%]) or recreational (14.2% [95% CI, 12.9%-15.5%] vs 11.3% [95% CI, 10.0%-12.6%]; difference, 2.9% [95% CI, 1.0%-4.7%]) purposes. After adjusting for covariates, adults with medical conditions had a higher prevalence of current marijuana use than those without medical conditions across all subgroups.

The prevalence of current and daily marijuana use decreased with increasing age (eFigure in the Supplement). Across age groups, adults who reported medical conditions had a higher prevalence of current and daily marijuana use than those without medical conditions except for those aged 65 years or older. For instance, among those aged 18 to 24 years, the prevalence of current (25.2% [95% CI, 22.0%-28.3%] vs 14.2% [95% CI, 12.7%-15.8%]; difference 10.9% [95% CI, 7.4%-14.4%]) and daily (11.2% [95% CI, 8.7%-13.6%] vs 5.3% [95% CI, 4.4%-6.2%]; difference, 5.9% [95% CI, 3.2%-8.5%]) marijuana use was much higher for those with medical conditions than those without medical conditions. Among those aged 65 years and older with medical conditions, the prevalence was 2.4% (95% CI, 2.0%-2.8%) for current marijuana use and 0.9% (95% CI, 0.7%-1.2%) for daily marijuana use.

The state-specific prevalence of marijuana use by age group and medical condition is presented in the Figure. There was a large variation in marijuana use across US states and territories. For instance, Alaska had the highest prevalence of current marijuana use among adults aged 18 to 34 years with medical conditions (38%; 95% CI, 30.1%-46.7%), more than 4 times higher than the prevalence among their counterparts in Guam (9%; 95% CI, 2.1%-16.3%).

**Figure.**
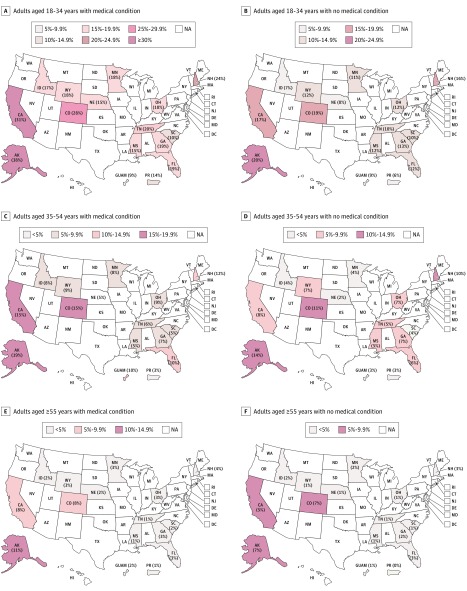
Prevalence of Current Marijuana Use by Select State and Medical Conditions, Combined 2016 and 2017 Behavioral Risk Factor Surveillance System Surveys Maps show percentage of current marijuana use among adults in the following categories: aged 18 to 34 years with a medical condition (A), aged 18 to 34 years without a medical condition (B), aged 35 to 54 years with a medical condition (C), aged 35 to 54 years without a medical condition (D), aged 55 years and older with a medical condition (E), and aged 55 years and older without a medical condition (F). At the time of the survey, medical marijuana use was legal in Minnesota, and recreational was legal in Alaska, Colorado, and California. NA indicates not applicable.

Table 2 presents the prevalence and adjusted odds ratios (AORs) of current marijuana use by age group and medical condition. Across age groups, adults who reported any medical condition had higher odds of current marijuana use than those who reported no medical conditions (AORs, 1.8 [95% CI, 1.5-2.1] for adults aged 18-34 years, 1.4 [95% CI, 1.2-1.7] for adults aged 35-54 years, and 1.6 [95% CI, 1.3-2.0] for adults aged ≥55 years). Adults with multiple medical conditions were more likely to report current marijuana use than those with only 1 medical condition. Among young adults aged 18 to 34 years, ever having a stroke (AOR, 2.5; 95% CI, 1.2-5.1), heart attack (AOR, 2.7; 95% CI, 1.2-6.1), asthma (AOR, 1.6; 95% CI, 1.3-1.9), COPD (AOR, 3.4; 95% CI, 2.3-4.9), arthritis (AOR, 2.0; 95% CI, 1.6-2.6), cancer other than skin cancer (AOR, 2.8; 95% CI, 1.7-4.6), or depression (AOR, 2.8; 95% CI, 2.3-3.4) was associated with higher odds of reporting current marijuana use compared with those without chronic medical conditions. Among adults aged 35 to 54 years, ever having asthma (AOR, 1.5; 95% CI, 1.2-2.0), COPD (AOR, 1.8; 95% CI, 1.3-2.5), arthritis (AOR, 1.5; 95% CI, 1.2-1.8), kidney disease (AOR, 2.7; 95% CI, 1.3-5.7), cancer other than skin cancer (AOR, 2.2; 95% CI, 1.6-3.2), or depression (AOR, 2.0; 95% CI, 1.6-2.5) was associated with increased odds of current marijuana use. Among adults aged 55 years and older, ever having a heart attack (AOR, 1.7; 95% CI, 1.2-2.5), coronary heart disease (AOR, 1.6; 95% CI, 1.1-2.2), asthma (AOR, 2.1; 95% CI, 1.5-2.8), COPD (AOR, 2.1; 95% CI, 1.6-2.9), arthritis (AOR, 1.5; 95% CI, 1.2-1.9), kidney disease (AOR, 1.7; 95% CI, 1.0-2.9), skin cancer (AOR, 1.5; 95% CI, 1.1-2.1), other cancer (AOR, 1.9; 95% CI, 1.4-2.8), or depression (AOR, 2.8; 95% CI, 2.3-3.4) was associated with higher odds of reporting current marijuana use compared with those without chronic medical conditions.

**Table 2.** Prevalence of Current Marijuana Use by Age Group and Medical Condition, Combined 2016 and 2017 Behavioral Risk Factor Surveillance System Surveys

Characteristic	Aged 18-34 y (n = 24 739)		Aged 35-54 y (n = 45 373)		Aged ≥55 y (n = 98 924)	
	Weighted % (95% CI)^a^	AOR (95% CI)^b^	Weighted % (95% CI)^a^	AOR (95% CI)^b^	Weighted % (95% CI)^a^	AOR (95% CI)^b^
Medical condition						
No	13.1 (12.1-14.1)	1 [Reference]	6.5 (5.7-7.3)	1 [Reference]	3.0 (2.5-3.5)	1 [Reference]
Any	21.9 (20.2-23.7)	1.8 (1.5-2.1)	9.9 (8.8-10.9)	1.4 (1.2-1.7)	4.1 (3.7-4.5)	1.6 (1.3-2.0)
No. of medical conditions						
1	21.1 (19.0-23.1)	1.9 (1.6-2.2)	8.2 (6.9-9.5)	1.3 (1.0-1.6)	3.5 (2.9-4.0)	1.3 (1.0-1.6)
2	23.8 (19.7-27.9)	2.1 (1.6-2.7)	11.6 (9.7-13.4)	1.9 (1.5-2.4)	4.8 (3.9-5.7)	2.0 (1.5-2.7)
≥3	25.7 (19.5-31.9)	3.1 (2.0-4.9)	12.6 (9.7-15.5)	1.8 (1.4-2.3)	4.1 (3.5-4.8)	1.8 (1.4-2.4)
Type of medical condition						
Stroke	22.4 (10.8-34.0)	2.5 (1.2-5.1)	8.6 (5.1-12.1)	1.0 (0.6-1.7)	3.9 (2.9-4.9)	1.5 (1.0-2.2)
Heart attack	26.9 (13.2-40.6)	2.7 (1.2-6.1)	11.6 (7.2-16.0)	1.6 (1.0-2.5)	3.8 (2.8-4.8)	1.7 (1.2-2.5)
Angina or coronary heart disease	11.2 (4.1-18.3)	1.2 (0.5-2.8)	8.6 (4.9-12.4)	1.3 (0.8-2.2)	3.8 (2.8-4.8)	1.6 (1.1-2.2)
Asthma	19.8 (17.3-22.2)	1.6 (1.3-1.9)	10.9 (8.6-13.1)	1.5 (1.2-2.0)	5.2 (4.1-6.3)	2.1 (1.5-2.8)
Chronic obstructive pulmonary disease	30.7 (24.0-37.3)	3.4 (2.3-4.9)	13.0 (10.3-15.7)	1.8 (1.3-2.5)	5.1 (4.1-6.1)	2.1 (1.6-2.9)
Diabetes	11.4 (6.1-16.7)	0.9 (0.5-1.7)	5.2 (3.7-6.7)	0.8 (0.6-1.2)	2.3 (1.7-2.9)	1.0 (0.7-1.3)
Arthritis	20.2 (16.7-23.7)	2.0 (1.6-2.6)	10.2 (8.6-11.8)	1.5 (1.2-1.8)	3.5 (3.1-4.0)	1.5 (1.2-1.9)
Kidney disease	20.0 (9.7-30.3)	1.4 (0.6-3.2)	14.3 (6.3-22.3)	2.7 (1.3-5.7)	4.1 (2.4-5.9)	1.7 (1.0-2.9)
Skin cancer	14.8 (2.9-26.7)	1.5 (0.5-4.4)	10.5 (4.3-16.7)	1.3 (0.8-1.9)	3.9 (3.1-4.8)	1.5 (1.1-2.1)
Other cancer	23.7 (14.9-32.5)	2.8 (1.7-4.6)	11.3 (8.3-14.3)	2.2 (1.6-3.2)	4.4 (3.3-5.5)	1.9 (1.4-2.8)
Depression	26.7 (24.0-29.4)	2.8 (2.3-3.4)	12.7 (11.2-14.1)	2.0 (1.6-2.5)	6.9 (5.9-8.0)	2.8 (2.2-3.7)

Abbreviation: AOR, adjusted odds ratio.

^a^ Weighted percentages and 95% CIs were reported by taking the complex sampling design into account.

^b^ Multivariable logistic regression was performed to compare the difference in marijuana use by medical condition. The analysis was adjusted by age, sex, race/ethnicity, education, income, survey year, and state.

Table 3 presents the distribution of primary ways to use marijuana among current marijuana users. In 2017, most current marijuana users (77.5%; 95% CI, 74.7%-80.3%) reported that they primarily smoked marijuana, followed by eating it (9.0%; 95% CI, 7.0%-10.9%), vaporizing it (9.0%; 95% CI, 7.1%-10.9%), dabbing it (3.1%; 95% CI, 2.0%-4.2%), other ways (1.1%; 95% CI, 0.6%-1.6%), and drinking it (0.4%; 95% CI, 0.1%-0.7%). Daily marijuana users were more likely than nondaily marijuana users to report smoking marijuana (83.0% [95% CI, 79.3%-86.7%] vs 72.8% [95% CI, 68.7%-76.8%]) and less likely to report eating marijuana (4.2% [95% CI, 2.4%-5.9%] vs 13.0% [95% CI, 9.9%-16.2%]). The method of administration was similar between adults with and without medical conditions.

**Table 3.** Distribution of Ways to Primarily Use Marijuana Among Current Marijuana Users, 2017 Behavioral Risk Factor Surveillance System Surveys

Characteristic	Ways to Primarily Use Marijuana, Weighted % (95% CI)^a^					
	Smoke (n = 2750)	Eat (n = 265)	Vaporize (n = 214)	Drink (n = 23)	Dab (n = 62)	Other (n = 47)
Overall	77.5 (74.7-80.3)	9.0 (7.0-10.9)	9.0 (7.1-10.9)	0.4 (0.1-0.7)	3.1 (2.0-4.2)	1.1 (0.6-1.6)
Daily marijuana user						
No	72.8 (68.7-76.8)	13.0 (9.9-16.2)	10.6 (8-13.2)	0.4 (0.0-1.0)	2.0 (0.7-3.3)	1.2 (0.5-1.9)
Yes	83.0 (79.3-86.7)	4.2 (2.4-5.9)	7.0 (4.3-9.8)	0.4 (0.0-0.7)	4.4 (2.5-6.3)	1.0 (0.3-1.8)
Medical condition						
No	77.9 (73.7-82.2)	10.2 (6.8-13.5)	8.9 (6.2-11.6)	0.1 (0.0-0.1)	2.4 (1.1-3.7)	0.6 (0.0-1.2)
Any	77.1 (73.4-80.8)	8.0 (5.8-10.3)	9.0 (6.4-11.6)	0.7 (0.1-1.3)	3.7 (2.0-5.4)	1.5 (0.7-2.3)
No. of medical conditions						
1	77.7 (72.4-83.0)	7.9 (4.8-11.0)	9.4 (5.4-13.4)	0.1 (0.0-0.3)	4.2 (1.7-6.7)	0.7 (0.1-1.4)
2	79.2 (72.9-85.6)	6.1 (2.8-9.3)	9.5 (5.1-14.0)	1.5 (0.0-3.5)	2.9 (0.0-6.0)	0.8 (0.1-1.5)
≥3	72.3 (64.1-80.5)	11.2 (4.9-17.6)	7.2 (2.4-12.1)	1.1 (0.0-2.4)	3.3 (0.0-6.6)	4.8 (1.2-8.5)
Type of medical condition						
Stroke	88.3 (79.5-97.1)	5.0 (0.0-11.7)	1.2 (0.0.4-2.1)	0.5 (0.0-1.3)	2.5 (0.0-7.1)	2.4 (0.0-5.6)
Heart attack	88.3 (77.2-99.4)	1.7 (0.0-4.4)	1.0 (0.0-2.1)	0.0 (0.0-0.0)	1.9 (0.0-5.6)	7.1 (0.0-17.4)
Angina or coronary heart disease	88.6 (81.7-95.5)	2.6 (0.0-5.4)	2.1 (0.0-4.5)	0.2 (0.0-0.6)	3.0 (0.0-6.9)	3.5 (0.0-7.1)
Asthma	75.7 (69.6-81.8)	7.9 (4.4-11.3)	9.7 (5.2-14.2)	1.4 (0.0-3.1)	2.6 (0.5-4.7)	2.6 (0.6-4.7)
Chronic obstructive pulmonary disease	82.5 (74.2-90.9)	3.5 (0.5-6.6)	4.5 (0.0-9.4)	0.7 (0.0-2.1)	5.3 (0.0-10.5)	3.5 (0.0-7.8)
Diabetes	80.6 (68.4-92.8)	8.5 (0.0-18.8)	3.0 (0.2-5.7)	0.1 (0.0-0.2)	4.6 (0.0-11.5)	3.3 (0.0-6.9)
Arthritis	69.9 (63.5-76.3)	14.2 (8.9-19.6)	9.1 (5.0-13.1)	1.2 (0.1-2.4)	2.4 (0.6-4.2)	3.1 (0.8-5.4)
Kidney disease	68.6 (50.6-86.6)	1.2 (0.0-3.4)	15.1 (1.6-28.5)	1.6 (0.0-4.7)	10.1 (0.0-23.7)	3.5 (0.0-7.4)
Skin cancer	68.3 (53.4-83.2)	15.1 (2.1-28.1)	5.8 (0.2-11.5)	1.8 (0.0-5.1)	7.6 (0.0-16.3)	1.4 (0.0-3.4)
Other cancer	65.9 (52.5-79.3)	10.6 (3.1-18.0)	16.5 (4.3-28.6)	1.1 (0.0-2.6)	3.4 (0.0-9.9)	2.6 (0.0-5.7)
Depression	79.2 (74.5-83.9)	6.6 (3.9-9.3)	8.3 (5.2-11.5)	0.7 (0.0-1.6)	3.5 (1.3-5.7)	1.7 (0.5-3.0)

^a^ Weighted percentages and 95% CIs were reported by taking the complex sampling design into account.

As shown in Table 4, in 2017, 35.1% (95% CI, 31.9%-38.2%) of current marijuana users reported that they used marijuana only for medical reasons, 45.6% (95% CI, 42.4%-48.9%) reported that they used marijuana only for nonmedical purposes, and 19.3% (95% CI, 16.3%-22.2%) reported they used marijuana for both reasons. Daily marijuana users were less likely than nondaily users to report using marijuana for nonmedical purposes (35.6% [95% CI, 30.8%-40.3%] vs 53.6% [95% CI, 49.1%-58.1%]) but more likely to report using marijuana for both reasons (26.5% [95% CI, 21.9%-31.1%] vs 13.6% [9.7%-17.4%]; *P* < .001). Adults with medical conditions were more likely than those without medical conditions to report using marijuana for medical reasons (45.5% [95% CI, 41.1%-49.8%] vs 21.8% [95% CI, 17.8%-25.7%]; difference, 23.7% [95% CI, 17.8%-29.6%]) and less likely to report using marijuana for nonmedical reasons (36.2% [95% CI, 32.1%-40.3%] vs 57.7% [95% CI, 52.6%-62.9%]; difference, −21.5% [95% CI, −28.1% to 14.9%]) or for both reasons (18.3% [95% CI, 14.8%-21.8%] vs 20.5% [95% CI, 15.6%-25.5%]). The likelihood of reporting use of marijuana for medical purposes increased by the number of medical conditions (36.1% [95% CI, 30.0%-42.1%] for 1 medical condition vs 68.7% [95% CI, 60.6%-76.8%] for ≥3 conditions; *P* < .001). Reasons for using marijuana also varied by type of medical condition. For example, among those using marijuana for medical purposes, the prevalence ranged from 64.3% (95% CI, 57.6%-70.9%) for those with arthritis to 43.7% (95% CI, 33.8%-53.6%) for those with COPD.

**Table 4.** Distribution of Reasons to Use Marijuana Among Current Marijuana Users, 2017 Behavioral Risk Factor Surveillance System Survey

Characteristic	Reasons to Use Marijuana, Weighted % (95% CI)^a^		
	Medical (n = 1217)	Nonmedical (n = 1763)	Both (n = 606)
Overall	35.1 (31.9-38.2)	45.6 (42.4-48.9)	19.3 (16.3-22.2)
Daily marijuana user			
No	32.8 (28.6-37.1)	53.6 (49.1-58.1)	13.6 (9.7-17.4)
Yes	37.9 (33.2-42.7)	35.6 (30.8-40.3)	26.5 (21.9-31.1)
Medical condition			
No	21.8 (17.8-25.7)	57.7 (52.6-62.9)	20.5 (15.6-25.5)
Any condition	45.5 (41.1-49.8)	36.2 (32.1-40.3)	18.3 (14.8-21.8)
No. of medical conditions			
1	36.1 (30.0-42.1)	44.1 (38.1-50.1)	19.8 (14.4-25.3)
2	46.7 (38.8-54.6)	35.7 (27.8-43.6)	17.6 (11.6-23.7)
≥3	68.7 (60.6-76.8)	16.2 (10.1-22.3)	15.1 (9.4-20.8)
Type of medical condition			
Stroke	54.3 (37.7-71.0)	30.2 (15.2-45.3)	15.4 (4.2-26.7)
Heart attack	49.8 (32.3-67.4)	37.8 (20.5-55.1)	12.3 (0.0-24.7)
Angina or coronary heart disease	52.6 (35.9-69.3)	29.7 (13.7-45.8)	17.7 (3.8-31.6)
Asthma	45.9 (38.1-53.7)	37.1 (30.0-44.1)	17.0 (12.0-22.1)
Chronic obstructive pulmonary disease	43.7 (33.8-53.6)	33.7 (23.8-43.7)	22.6 (13.6-31.6)
Diabetes	51.7 (38.0-65.4)	33.0 (19.6-46.4)	15.3 (6.4-24.2)
Arthritis	64.3 (57.6-70.9)	23.1 (17.1-29.1)	12.7 (9.0-16.3)
Kidney disease	57.4 (36.3-78.6)	24.2 (6.5-41.8)	18.4 (4.7-32.0)
Skin cancer	62.5 (46.5-78.4)	27.2 (14.3-40.0)	10.4 (2.1-18.6)
Other cancer	47.7 (35.5-59.9)	22.9 (12.3-33.5)	29.4 (16.3-42.5)
Depression	48.9 (43.2-54.5)	31.7 (26.4-37.0)	19.4 (14.3-24.6)

^a^ Weighted percentages and 95% CIs were reported by taking the complex sampling design into account.

## Discussion

To our knowledge, this is the first study to report national estimates of current and daily marijuana use among adults with medical conditions. Compared with those with no medical conditions, adults with medical conditions had a significantly higher prevalence of current and daily marijuana use across all age groups except those aged 65 years or older. Among young adults aged 18 to 24 years with medical conditions, 25.2% reported current use of marijuana and 11.2% used marijuana on a daily basis.

Confidence in our findings is bolstered by the fact that our findings of marijuana use among the general population are consistent with previous studies.^10^ We found that marijuana use decreased with increasing age and that adults aged 18 to 24 years had the highest prevalence of current marijuana use (25.2%), more than 10 times higher than those aged 65 years and older (2.4%). We also found a wide variation across age groups associated with marijuana use among adults with and without medical conditions. These disparities could be due to differences in perceived harm and benefits of marijuana use across age groups. For instance, Keyhani et al^4^ reported that adults aged 65 years or older were more likely to view marijuana as very addictive and harmful compared with adults in other age groups, partly because of the stigma in past decades that was associated with marijuana use. However, public opinions on marijuana use have been softening,^1^ and fewer older adults are reporting disapproval of marijuana use.^11^ The increase in public acceptance of marijuana use could lead older adults to start using marijuana for medical conditions.

This study also identified a large variation of marijuana use among adults with medical conditions across select US states and territories, with Alaska having a prevalence among those aged 18 to 34 years more than 4 times higher than that in Guam (38% vs 9%). The geographic variation in marijuana use could be attributable to the availability of marijuana products associated with legal status as well as variations in perceptions of the risks and benefits of marijuana.

Consistent with previous studies,^12,13^ this analysis found that combusted methods of marijuana administration were most prevalent among US adults. We add to the literature by determining that the method of administration did not differ between adults with and without medical conditions. It is of concern that the great majority (77.5%) of current marijuana users with medical conditions consume marijuana by smoking it. Marijuana smoke contains many chemicals found in cigarette smoke (eg, carcinogens, carbon monoxide, tar, and bronchial irritants)^14,15^ and is associated with adverse outcomes on pulmonary function and increased respiratory symptoms.^15,16^

Nearly one-half (45.5%) of people with medical conditions reported that they use marijuana solely for medical purposes. They were more likely to report this than people with no medical conditions. However, most people with medical conditions were using marijuana recreationally—for only nonmedical purposes (36.2%) and for both medical and nonmedical reasons (18.3%). In scenarios where a drug has well-established health benefits, good manufacturing processes, and minimal risks, the reason that a patient takes a drug is immaterial. For example, it does not matter that a person with migraine headaches, who also has concerns about aging skin, derives both medical and cosmetic benefits from Botox therapy. Marijuana, however, can vary widely according to mode of delivery and is associated with several known adverse health outcomes,^5,17^ including increased respiratory symptoms, impaired short-term memory, and increased risk of psychiatric illness and addiction. It is important for health care professionals to understand whether patients are using marijuana for medical or recreational purposes, and how patients are consuming their marijuana, to better advise patients about the adverse health outcomes and potential benefits.

Patients who are using marijuana for a medical condition should be informed of evidence of efficacy and adverse outcomes for that condition. Those who are using marijuana recreationally should also be informed of the adverse health outcomes and benefits of marijuana consumption. Clinical information that might facilitate such patient-clinician discussions includes use and frequency of marijuana, use and adherence to evidence-based treatment for the chronic condition(s) of the patient, the patient’s chronic disease control and outcomes, and marijuana-related health outcomes and symptoms.

This study further examined the associations between marijuana use and 11 chronic health conditions. It is notable that adults with respiratory conditions, such as asthma or COPD, reported a higher prevalence of current marijuana use than those without these conditions across all age groups. For instance, adults aged 18 to 34 years with COPD had almost 3 times higher odds of reporting current marijuana use than their peers without COPD (AOR, 3.4; 95% CI, 2.3-4.9). It is possible that long-term marijuana use was a contributing factor to their comorbid condition. It is also possible that these adults were using marijuana for relief from pain, anxiety, stress, or depression.^4^

The benefits of marijuana in treating chronic pain have been documented in previous studies.^18,19^ A report from the National Academies of Sciences, Engineering, and Medicine^19^ concluded that there is substantive evidence that cannabis is effective for the treatment of chronic pain in adults. One systemic study^18^ reviewed 16 studies with 1750 participants and found that the potential benefits of cannabis-based medicine in chronic neuropathic pain might be outweighed by their potential harms. However, another systemic review^20^ of intervention trials and observational trials also found limited or insufficient evidence that marijuana use may relieve neuropathic pain or other types of chronic pain, and findings on marijuana’s effects on anxiety, stress, and depression are mixed to negative.^21^ This suggests that some patients who are using marijuana for these conditions could be better served by evidence-based psychotherapy or US Food and Drug Administration–approved medications than by continued use of marijuana. Primary care clinicians and specialists should be aware that patients in their care with medical conditions, especially young adults, have a high probability of marijuana use. They should screen for marijuana use and have open conversations regarding the potential adverse health effects and benefits of marijuana use for patients’ specific conditions.

### Limitations

Our findings are subject to several limitations. First, the BRFSS data are cross-sectional; thus, we were unable to examine causal relationships between medical comorbidity and marijuana use. Second, data were collected from select US states. The BRFSS supported 25 modules in 2016 and 29 modules in 2017, but states limited modules to only the most useful for their state program purposes to keep surveys at a reasonable length.^7^ Thus, our findings were limited to these states and might not be generalizable to populations residing in other states or the United States as a whole. However, the prevalence estimates of current marijuana use among adults with medical conditions (8.8%) and those without medical conditions (8.3%) in our study are close to the results of another national study (prevalence, 8.7%) using KnowledgePanel.^12^ Third, chronic health conditions are self-reported and are subject to social desirability bias. However, the reliability and validity of BRFSS data on medical conditions have been validated in previous studies.^22,23,24^ Fourth, because the BRFSS only includes questionnaires regarding lifetime status of most chronic health conditions (“Has a doctor, nurse, or other health professional ever told you that you have…?”), we were unable to measure the current status of chronic health conditions.

## Conclusions

This study analyzed a large probability sample about current and daily marijuana use among US adults with medical conditions and reported large variations of marijuana use by key sociodemographic factors and geographic regions. Adults with medical conditions, especially those with respiratory conditions, cancer, and depression, were more likely to use marijuana. At present, marijuana use prevalence decreases with age, even among people with medical conditions. Because public perceptions of marijuana are becoming more favorable and medical conditions increase with age, older adults might also become frequent consumers of marijuana. Hence, continuous surveillance of marijuana use across all age groups is warranted. Clinicians should screen for marijuana use among patients and initiate open discussions with patients about the benefits and risks associated with marijuana for their comorbid conditions and long-term health. Policy makers should monitor health claims made by merchants and perceptions of benefits among consumers to ensure that patients—especially those with existing medical conditions—understand the evolving knowledge base regarding the risks and benefits of marijuana consumption.

## Notes

Supplement. **eTable.** Sociodemographic Comparison of Adults With and Without Medical Conditions, Combined 2016 and 2017 BRFSS **eFigure.** Prevalence of Current and Daily Marijuana Use by Medical Condition and Age Group
