# Supplementary material for: A National Survey of Marijuana Use Among US Adults With Medical Conditions, 2016-2017
Source: JAMA Netw Open. 2019 Sep 20;2(9):e1911936. doi: 10.1001/jamanetworkopen.2019.11936 (PMC6755533; doi:10.1001/jamanetworkopen.2019.11936)
Supplement: Supplement. — eTable. Sociodemographic Comparison of Adults With and Without Medical Conditions, Combined 2016 and 2017 BRFSS eFigure. Prevalence of Current and Daily Marijuana Use by Medical Condition and Age Group [file jamanetwopen-2-e1911936-s001.pdf]

Supplementary Online Content

Dai H, Richter KP. A national survey of marijuana use among US adults with medical conditions, 2016-2017. *JAMA Netw Open*. 2019;2(9):e1911936. doi:10.1001/jamanetworkopen.2019.11936

eTable. Sociodemographic Comparison of Adults With and Without Medical Conditions, Combined 2016 and 2017 BRFSS

eFigure. Prevalence of Current and Daily Marijuana Use by Medical Condition and Age Group

This supplementary material has been provided by the authors to give readers additional information about their work.

© 2019 Dai H et al. *JAMA Network Open.*

eTable. Sociodemographic Comparison of Adults With and Without Medical Conditions, Combined 2016 and 2017 BRFSS[a]

			Lifetime Diagnosis of Chronic Health Conditions				
			No medical condition		Any medical condition		-
Characteristics	n	Weighted % (95% CI)[b]	n	Weighted % (95% CI)[b]	n	Weighted % (95% CI)[b]	p-value[c]
Overall	169,036	100	64,808	46.3 (45.8-46.9)	104,228	53.7 (53.1-54.2)	
Year							0.0407
2016	106,067	45.8 (45.5-46.2)	40,341	45.2 (44.5-45.9)	65,726	46.3 (45.7-46.9)	
2017	62,969	54.2 (53.8-54.5)	24,467	54.8 (54.1-55.5)	38,502	53.7 (53.1-54.3)	
Age							<.0001
18-24	8,637	11.5 (11.1-11.9)	5,770	16.5 (15.8-17.3)	2,867	7.1 (6.6-7.6)	
25-34	16,102	15.9 (15.4-16.3)	10,476	22.8 (22.0-23.5)	5,626	9.9 (9.5-10.4)	
35-44	19,121	16.2 (15.8-16.7)	11,397	21.2 (20.5-21.9)	7,724	11.9 (11.4-12.4)	
45-54	26,252	17.0 (16.6-17.5)	12,506	17.7 (17.0-18.3)	13,746	16.5 (15.9-17.1)	
55-64	37,554	17.4 (17.0-17.8)	12,708	12.7 (12.2-13.3)	24,846	21.4 (20.8-22.0)	
65+	61,370	22.0 (21.6-22.4)	11,951	9.1 (8.7-9.5)	49,419	33.1 (32.5-33.8)	
Sex							<.0001
Male	73,234	48.0 (47.5-48.6)	31,502	52.5 (51.6-53.4)	41,732	44.2 (43.4-44.9)	
Female	95,780	52.0 (51.4-52.5)	33,300	47.5 (46.6-48.4)	62,480	55.8 (55.1-56.6)	
Race/Ethnicity							<.0001
Non-Hispanic white	129,834	60.9 (60.4-61.5)	46,684	53.1 (52.0.3-54)	83,150	67.6 (66.9-68.4)	
Non-Hispanic black	12,184	11.0 (10.7-11.4)	4,976	12 (11.4-12.5)	7,208	10.2 (9.8-10.7)	
Hispanic	15,778	19.9 (19.4-20.4)	8,185	24.8 (24.0-25.6)	7,593	15.7 (15.1-16.3)	
Others	8,518	8.2 (7.7-8.6)	3,912	10.1 (9.4-10.9)	4,606	6.5 (5.9-7.0)	
Education							<.0001
Less than high school	13,404	14.3 (13.9-14.8)	4,226	13.1 (12.4-13.8)	9,178	15.4 (14.8-16.0)	
High school graduate	47,415	27.5 (27.1-28.0)	16,901	27.3 (26.5-28.0)	30,514	27.8 (27.2-28.4)	
Some college	48,709	32.2 (31.6-32.7)	17,982	30.8 (29.9-31.6)	30,727	33.4 (32.7-34.1)	
College graduate	58,980	26.0 (25.5-26.4)	25,460	28.9 (28.2-29.6)	33,520	23.4 (22.9-24.0)	

© 2019 Dai H et al. *JAMA Network Open.*

Income							<.0001
<25,000	40,671	29.7 (29.1-30.2)	11,570	25 (24.2-25.8)	29,101	33.7 (33-34.4)	
25,000-50,000	37,168	23.6 (23.1-24.1)	13,507	22.9 (22.2-23.7)	23,661	24.2 (23.5-24.9)	
50,000-75,000	22,886	14.8 (14.3-15.2)	9,539	15.3 (14.6-15.9)	13,347	14.4 (13.8-14.9)	
75,000+	43,270	32 (31.4-32.5)	21,079	36.9 (35.9-37.8)	22,191	27.7 (27-28.4)	
Employment Status							<.0001
Employed	81,992	55.8 (55.3-56.4)	43,377	69.8 (69-70.6)	38,615	43.8 (43.1-44.5)	
Unemployed	6,749	5.3 (5.0-5.5)	2,559	5.2 (4.8-5.6)	4,190	5.3 (5.0-5.6)	
Not in workforce	79,213	38.9 (38.4-39.4)	18,331	25.0 (24.2-25.8)	60,882	50.9 (50.1-51.6)	
Home ownership							<.0001
Own	125,597	68.2 (67.7-68.7)	46,274	63.9 (63.1-64.7)	79,323	71.9 (71.2-72.5)	
Rent	35,912	26.5 (26.0-27.0)	15,205	30 (29.2-30.8)	20,707	23.5 (22.9-24.1)	
Other arrangement	6,724	5.3 (5.1-5.5)	2,985	6.1 (5.7-6.5)	3,739	4.6 (4.3-4.9)	
Marijuana Legalization Status[d]							<.0001
No	114,491	58.9 (58.6-59.3)	41,534	56.7 (55.9-57.4)	72,957	60.9 (60.3-61.6)	
Medical	29,847	9.1 (9.0-9.2)	13,147	9.8 (9.6-10)	16,700	8.5 (8.4-8.7)	
Recreational	24,698	31.9 (31.5-32.3)	10,127	33.5 (32.7-34.4)	14,571	30.6 (29.8-31.3)	

[a] In 2016,10 states (Alaska, Colorado, Florida, Idaho, Minnesota, Mississippi, Nebraska, Ohio, Tennessee, Wyoming) participated in the optional "marijuana use" module. In 2017, 9 states (Alaska, California, Georgia, Idaho, Minnesota, New Hampshire, South Carolina, Tennessee, and Wyoming) and 2 territories (Guam and Puerto Rico) participated in the optional "marijuana use" module.

[b]Weighted % and 95% CI were reported by taking the complex sampling design into account.

[c]A Rao-Scott Chi-square test was performed to detect the group difference.

[d]In 2016, medical legalization: Minnesota; recreational legalization: Alaska and Colorado. In 2017, medical legalization: Minnesota; recreational legalization: Alaska and California.

© 2019 Dai H et al. *JAMA Network Open.*

eFigure. Prevalence of Current and Daily Marijuana Use by Medical Condition and Age Group

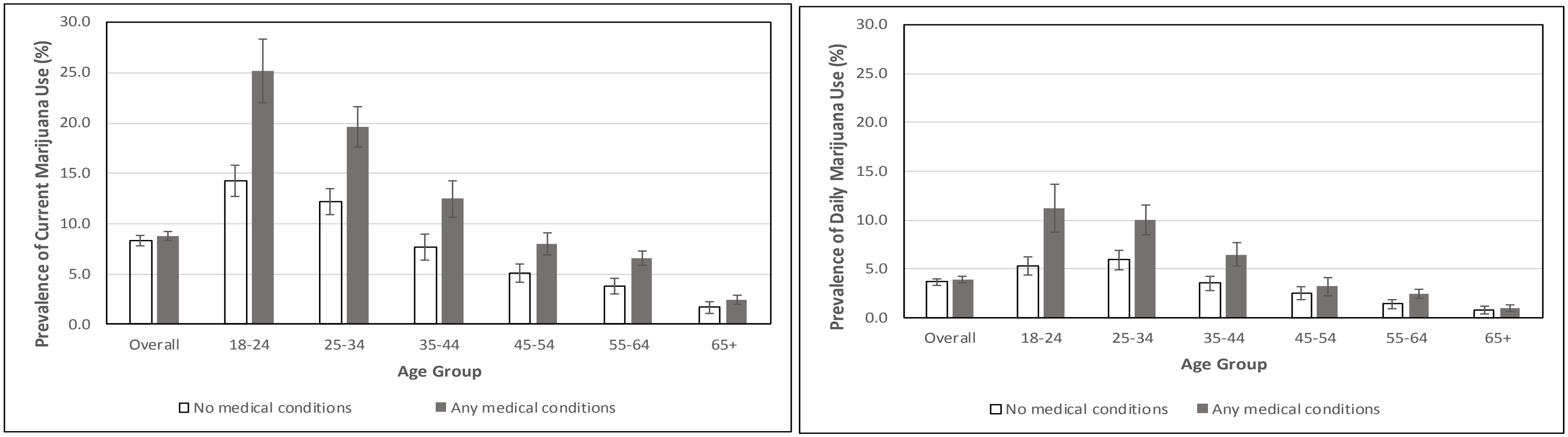

Graphs show prevalence and 95% CI of current marijuana use (left) and prevalence and 95% CI of daily marijuana use (right). Weighted % and 95% CI were reported by taking the complex sampling design into account. Error bars indicator 95% CI.

© 2019 Dai H et al. *JAMA Network Open.*
